# Hospital Dresser's Table

**Published:** 1893-03-18

**Authors:** 


### PRACTICAL DEPARTMENTS.

### HOSPITAL DRESSER'S TABLE.

Mr. Alfred Carter, 47, Holborn Viaduct, has recently brought out a new dresser's table, which may fairly claim to be an improvement upon those generally in use in hospital wards. It is marble-topped, with ledges running round and across, and a towel-rail at the back, and is provided with a sliding slab for preparing dressings, and two drawers, each divided into four compartments. These drawers run right through the table from end to end, so that their contents can be reached with equal facility from either side. There is also a bottom shelf for antiseptics, sponges, &c. The table is made in polished in birch, and being mounted on large brass castors, can be moved about from bed to bed with perfect ease. It will be found most useful in any surgical ward. We give below a sketch of this very convenient addition to ward resources.

